# Harnessing Artificial Intelligence in Interventional Cardiology: A Systematic Review of Current Applications

**DOI:** 10.7759/cureus.87494

**Published:** 2025-07-07

**Authors:** Priyansh Patel, Besiki Davitashvili, Sai Sujana Chitturi, Mari Gadaevi, Diya Patel, Jayanth Reddy Tallapalli, Jyothsna Butchireddy, Ria Suresh, Jayanth Jhishnu Nannegari, Shivam Slathia

**Affiliations:** 1 Department of Cardiovascular Medicine, University of Miami Miller School of Medicine, Miami, USA; 2 Department of Internal Medicine, Medical College, Baroda, Vadodara, IND; 3 Department of Internal Medicine, Tbilisi State Medical University, Tblisi, GEO; 4 Department of Cardiology, Dr. D. Y. Patel Medical College, Navi Mumbai, IND; 5 General Medicine, Gujarat Medical Education and Research Society (GMERS) Sola, Ahmedabad, IND; 6 Department of Infectious Diseases, University of South Florida, Tampa, USA; 7 Department of Medicine, Government Medical College Omandurar, Chennai, IND; 8 Department of Medicine, Government Kilpauk Medical College, Chennai, IND; 9 Department of Medicine, Bhaskar Medical College, Hyderabad, IND; 10 Department of Medicine, Acharya Shri Chander College of Medical Sciences, Jammu, IND

**Keywords:** ai and machine learning, ai & robotics healthcare, artificial intelligence (ai), cognitive computing, convolutional neural networks (cnn), deep learning (dl), interventional cardiology, natural language processing (nlp)

## Abstract

Interventional cardiology has recently advanced with innovations such as percutaneous transluminal coronary angioplasty (PTCA), transcatheter aortic valve replacement (TAVR), and the emergence of artificial intelligence (AI) as a transformative tool. This systematic review explored the current landscape, methodologies, and applications of AI in interventional cardiology. A comprehensive literature search was conducted following preferred reporting guidelines, identifying 20 studies after data extraction and quality assessment. AI-particularly machine learning (ML) and deep learning (DL)-enhances diagnostic accuracy and procedural efficiency. ML aids in arrhythmia detection and coronary plaque characterization, while DL supports imaging interpretation, robotic navigation, and catheter tracking. Clinical applications show AI's potential in predicting myocardial infarction, guiding personalized treatment, and improving resource management. Despite these benefits, challenges such as data privacy, algorithm transparency, and generalizability remain. Addressing these requires collaborative efforts and robust data sharing. Future priorities include integrating AI into routine clinical workflows, resolving regulatory barriers, and ensuring interpretability. Multidisciplinary collaboration is essential to address ethical considerations and uphold patient safety. The integration of AI in interventional cardiology offers significant potential to enhance patient care, procedural precision, and resource utilization. However, its adoption must be guided by careful attention to ethical, technical, and regulatory constraints. Overcoming these barriers through coordinated efforts may allow AI to redefine standards in cardiovascular care and enable a more precise, efficient, and patient-centered approach to interventional cardiology.

## Introduction and background

Interventional cardiology, a subspecialty focused on catheter-based treatment of cardiovascular diseases, has witnessed significant advancements - from the introduction of percutaneous transluminal coronary angioplasty (PTCA) to more complex procedures such as transcatheter aortic valve replacement (TAVR), robotic-assisted interventions, and advanced imaging modalities like intravascular ultrasound (IVUS), optical coherence tomography (OCT), and fractional flow reserve (FFR) [1]. In this evolving landscape, artificial intelligence (AI) has emerged as a transformative tool, reshaping clinical practice. By enhancing workflow efficiency, cost-effectiveness, and decision-making, AI holds substantial promise in cardiovascular medicine. AI refers to the use of computer systems to perform tasks traditionally requiring human intelligence. A key subfield, machine learning (ML), enables systems to learn from data and improve over time. Deep learning (DL), a subset of ML, utilizes artificial neural networks - particularly convolutional neural networks (CNNs) - to analyze complex medical images. Meanwhile, explainable AI (XAI) aims to make ML model outputs interpretable and transparent to human users [2].

AI has garnered considerable potential for refining the precision of imaging interpretation and for selecting the most efficacious transcatheter treatments. Currently, AI is widely utilized in robot-assisted surgery, risk assessment, and evaluation of transcatheter imaging quality [3]. The field of AI is progressing, presenting novel opportunities for healthcare professionals. However, the current utilization of AI in interventional cardiology is restricted by predefined outputs and ethical concerns, including patient privacy, data security, and potential algorithmic biases [3,4]. The implementation of AI in clinical practice has advanced from serving as a complementary tool in image analysis to playing an active role in therapeutic decision-making, resulting in enhanced patient care [5]. The AI’s capacity to not only assess intricate medical images but also contribute to decision-making processes emphasizes its multifaceted function in reshaping interventional cardiology. Furthermore, AI applications have demonstrated the potential to streamline workflows, decrease procedural times, and contribute to efficient resource utilization, which aligns with the broader goals of healthcare systems aiming for increased effectiveness and sustainability in a cost-effective manner for the administration and reward of patients [6]. Integrating AI into all aspects of delivering standard healthcare to a patient, from admission to determining the appropriate treatment method, is necessary. Therefore, the fusion of human expertise and AI capabilities holds promise for redefining the standards of patient care, procedural accuracy, and overall outcomes. This systematic review aimed to provide an extensive exploration of the current landscape, advantages, and potential future applications of AI in interventional cardiology, contributing to the ongoing dialogue on shaping the future of cardiovascular medicine, emphasizing its advantages over non-digital intervention practices, and shedding light on the potential future applications of AI in this field.

## Review

Methodology

Our systematic review was conducted in accordance with the preferred reporting guidelines for systematic reviews and meta-analyses (PRISMA) [7].

Search Strategy and Study Selection

Our search for relevant articles was conducted on databases like PubMed, Medline, PubMed Central, Cochrane, Embase, Scopus, and ClinicalTrials.gov using keywords such as “artificial intelligence”, “interventional cardiology”, “machine learning”, “cardiac catheterization”, “cardiology”, and “instrumentation”. “deep learning”, from their inception until February 29, 2024. All identified articles were imported into Zotero, and duplicates were removed. The initial screening of titles and abstracts was followed by full-text screening based on the predefined inclusion and exclusion criteria. Additionally, we manually searched the reference lists of all the included studies. Table 1 lists the search strategies and the number of papers identified in each database. Table 2 shows the eligibility criteria.

**Table 1:** Literature search PMC: PubMed Central

Database	Search Strategy	Paper Identified
PubMed/Medline/PMC keyword search	artificial intelligence AND interventional cardiology	283
	machine learning AND interventional cardiology	169
PubMed MeSH Search	("Machine Learning"[Majr]) AND ("Cardiac Catheterization"[Majr])	4
	("Machine Learning"[Majr]) AND (( "Cardiology/classification"[Majr] OR "Cardiology/instrumentation"[Majr] OR "Cardiology/methods"[Majr] ))	8
	("Artificial Intelligence"[Majr]) AND ("Cardiac Catheterization"[Majr])	37
	("Artificial Intelligence"[Majr]) AND (( "Cardiology/classification"[Majr] OR "Cardiology/instrumentation"[Majr] OR "Cardiology/methods"[Majr] ))	30
Cochrane Library	Artificial intelligence AND Cardiology	16
	Machine learning AND Cardiology	11
	Machine learning AND cardiac catheterization	4
	Artificial intelligence AND cardiac catheterization	5
Total		567

**Table 2:** Eligibility criteria

Inclusion Criteria	Exclusion Criteria
Papers in the English language	Papers in any language other than English.
Review articles, systematic reviews, meta-analyses, and observational studies are included.	Studies that cannot be retrieved as full text.
Studies with artificial intelligence or machine learning with respect to interventional cardiology or cardiac catheterization.	Studies discussing cardiology other than interventional aspects.
Full-text studies	Gray literature.
Studies from all times	Study types other than reviews, systematic reviews, meta-analyses, and observational studies.

Data Extraction

Data for the studies in the review were obtained using a pre-designed Microsoft Excel spreadsheet. Two authors were responsible for extracting the data, and two other authors cross-checked the extracted data for analysis. The extracted data included the author's name, year of publication, study design, and qualitative assessment of the article. Any discrepancies in the data extraction process were resolved through discussions among the authors.

Quality Appraisal of the Studies

The shortlisted articles were thoroughly evaluated for quality using established quality appraisal instruments. Two authors conducted quality checks, whereas another author performed cross-checks. In the event of any disagreements, all coauthors were involved in discussing the issues, and the final decision to accept or reject the paper was made through mutual consensus. Observational studies [8-11] were assessed using the Newcastle-Ottawa questionnaire. Review studies [12-27] were assessed using the scale for quality assessment of narrative review articles (SANRA) guidelines. These tools are primarily questionnaires regarding the methodology of the study, whose quality is being assessed. Only studies with an assessment of good quality were selected. In the Newcastle-Ottawa tool, a star is given to the study for each criterion met. Good quality: 3 or 4 stars in the selection domain AND 1 or 2 stars in the comparability domain AND 2 or 3 stars in the outcome domain. Fair quality: 2 stars in the selection domain AND 1 or 2 stars in the comparability domain AND 2 or 3 stars in the outcome domain. Poor quality: 0 or 1 star in the selection domain OR 0 stars in the comparability domain OR 0 or 1 stars in the outcome domain. The SANRA checklist assesses the quality from 0 (low standard) to 2 (high standard). Table 3 shows the quality assessment of the observational studies using the Newcastle-Ottawa Scale, and Table 4 shows the quality appraisal of review articles using the scale for quality assessment of narrative review articles (SANRA) checklist.

**Table 3:** Quality assessment of observational studies using the Newcastle-Ottawa tool In the Newcastle-Ottawa tool, a star is given to the study for each criterion met. Good quality: 3 or 4 stars in the selection domain AND 1 or 2 stars in the comparability domain AND 2 or 3 stars in the outcome domain. Fair quality: 2 stars in the selection domain AND 1 or 2 stars in the comparability domain AND 2 or 3 stars in the outcome domain. Poor quality: 0 or 1 star in the selection domain OR 0 stars in the comparability domain OR 0 or 1 stars in the outcome domain.

Study Title	Selection	Comparability	Outcome	Overall (Stars)
Min et al. [8]	***	*	***	Good (7)
Niedziela et al. [9]	***	*	***	Good (7)
Bezerra et al. [10]	****	*	****	Good (9)
D’Onofrio et al. [11]	***	*	***	Good (7)

**Table 4:** Quality assessment of review articles using SANRA checklist SANRA: Scale for quality assessment of narrative review articles.

Study Title	Justification of the Article’s Importance for Readership	Statement of Concrete/Specific Aims or Formulation of Questions	Description of Literature Search	Referencing	Scientific Reasoning	Appropriate Presentation of Data
Nagarajan et al. (2021) [12]	2	1	0	2	1	1
Gheorghe et al. (2021) [13]	2	1	0	2	1	0
Sardar et al. (2019) [14]	2	2	1	2	1	0
Seetharam et al. (2021) [15]	2	1	1	2	2	0
Samant et al. (2023) [16]	2	2	0	2	1	0
Molenaar et al. (2022) [17]	2	2	1	2	1	0
Seetharam et al. (2022) [18]	2	1	0	2	1	0
Ben Ali et al. (2021) [19]	2	2	0	2	1	0
Petousis et al. (2023) [20]	2	1	1	2	1	1
Chowdhury and Osborn [21]	2	1	0	2	1	0
Chu et al. (2023) [22]	2	2	1	2	1	0
Ramadani et al. (2022) [23]	2	1	0	2	1	1
Karako et al. (2019) [24]	2	1	1	2	0	1
Calvinho and Antunes (2008) [25]	1	1	1	1	0	0
Prandi et al. (2023) [26]	2	2	1	2	1	1
Kwiecinski et al. (2023) [27]	2	2	0	2	1	0

Results

Study Identification and Selection

**Figure 1:**
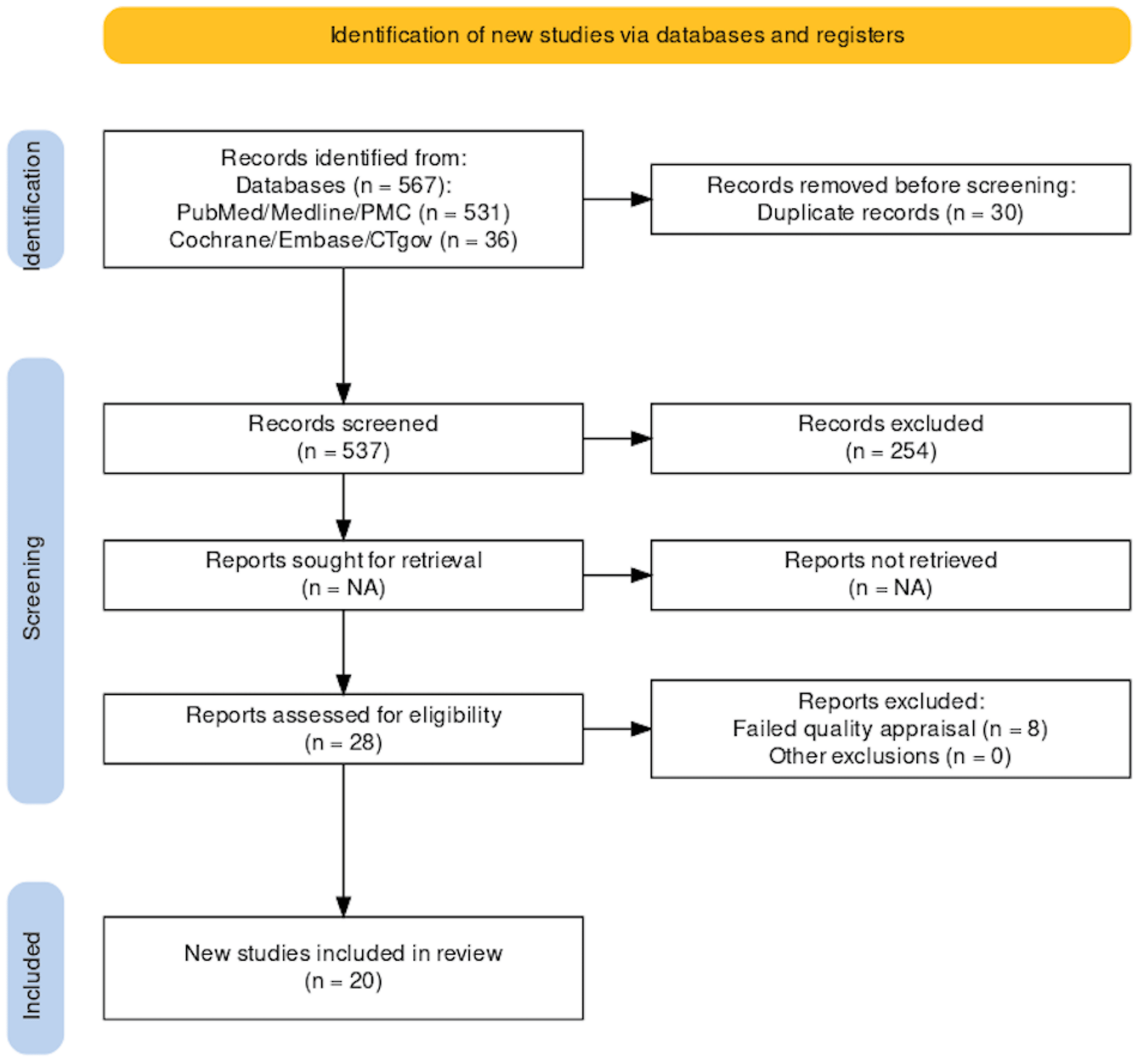
PRISMA flow diagram PRISMA: Preferred reporting items for systematic reviews and meta-analyses

We gathered a total of 567 relevant studies using all the databases. Of these, 254 were removed as they were not available as full text. Thirty duplicate studies were excluded before screening. After screening the remaining 283 studies based on titles, abstracts, and eligibility criteria, we were left with a shortlist of 28 studies. These shortlisted full-text articles were used for quality appraisal, and eight additional studies were removed, thus failing quality appraisal. The remaining 20 studies were used for the finalized review; four were observational studies and 16 were review articles. The selection process of finalized studies is shown in Figure 1 in the PRISMA flowchart [28]. Table 5 presents the characteristics and summary of the individual studies.

**Table 5:** Characteristics and summary of individual studies 3D: three dimensional; AC: self-expandable ACURATE-TA; ACS: acute coronary syndrome; AF: atrial fibrillation; AI: artificial intelligence; AISER: artificial intelligence computational simulations and extended reality; AKI: acute kidney injury; CAD: coronary artery disease; CMR: cardiac magnetic resonance; CNN: convoluted neural network; CT: computed tomography; DL: deep learning; ECG: electrocardiograph; EM: electromagnetic; FFR: fractional flow reserve; GAN: generative adversarial networks; HCM: hypertrophic cardiomyopathy; HF: heart failure; IC: interventional cardiology; ICA: invasive coronary angiography; iFR: instantaneous wave-free ratio; IVUS: intravascular ultrasound; LAD: left anterior descending; LGM: longitudinal geographic miss; LR: logistic regression; MACE: major adverse cardiovascular events; ML: machine learning; MRI: magnetic resonance imaging; NHPR: non-hyperemic pressure ratios; NLP: natural language processing; NN: neural network; OCT: optical coherence tomography; PCI: percutaneous coronary intervention; PET: positron emission tomography; PTCA: percutaneous transluminal coronary angioplasty; S3: sapien3; SPECT: single photon emission computed tomography; STEMI: ST elevation myocardial infarction; TA-TAVI: transapical transcatheter aortic valve implantation; TVR: target vessel revascularization; VT: ventricular tachycardia; VF: ventricular fibrillation

Author, Year	Country	Study Design	Tool for Data Analysis	Phase of the Process in Which AI is Engaged	Validation for Use	Main Results
Min et al., 2021 [8]	Korea	Observational Study	CNN	Pre-procedural IVUS phase	Yes	The study developed DL algorithms using pre-procedural IVUS images to predict stent under-expansion. The models demonstrated high accuracy (94%) in predicting incomplete stent expansion, aiding clinicians in identifying high-risk lesions and making treatment decisions to prevent stent failure.
Niedziela et al., 2021 [9]	Poland	Observational Study	NN	After the first STEMI	Yes	The study compared NN and LR for predicting 6-month mortality post-STEMI with LAD post-PCI. NN showed slightly better predictive value than LR, highlighting its potential for analyzing complex variables in predicting outcomes in STEMI patients. Further research is needed for clinical implementation.
Bezerra et al., 2015 [10]	United States	Observational Study	CorPath 200	The AI tool, CorPath 200 System, was engaged during the robotic-assisted PCI procedures, specifically in the phase of stent deployment.	Yes	The study compared robotic-assisted PCI to manual PCI. Robotic-assisted PCI demonstrated significantly lower LGM incidence than manual PCI, validated through propensity score matching. Benefits included reduced rates of MACE, TVR, and operator fatigue, enhancing procedural precision and patient outcomes. Limitations include patient differences and stent types.
D’Onofrio et al., 2023 [11]	Italy	Observational Study	ML model	The AI tool was engaged in the follow-up phase.	N/A	The study reviewed the use of ML for mortality prediction and evaluated valve durability. The study on TA-TAVI outcomes over 10 years showed good survival rates and valve durability. Comparing S3 and AC devices revealed similar outcomes, with low rates of complications and reoperations in both groups.
Nagarajan et al. 2019 [12]	China	Pilot study	ML model	Data analysis and interpretation in the process of arrhythmia detection and management	Yes	Research explores DL for MRI and wearable AF detection, transforming cardiology. The study demonstrated ML’s success in cardiac devices for arrhythmia detection, event prediction, and therapy. AI identified AF, improving cardiology management. Integration of AI-enhanced device functionality, transforming patient care. Collaboration and responsible AI use are vital.
Gheorghe et al., 2021 [13]	Multiple countries	Review	Computational modeling	Pre-procedural planning, intraprocedural guidance, and monitoring phases of the mitral valve interventions.	Yes	This technology uses imaging for therapy selection and outcome prediction. This technology offers detailed insights into mitral valve anatomy, enhancing procedural success and patient outcomes.
Sardar et al., 2019 [14]	United States	Review	ML model	To triage chest pain patients, in preoperative planning, and in predicting periprocedural risks such as death, bleeding, contrast nephropathy, and stroke	N/A	ML analyzes disease profiles; it predicts outcomes like contrast-induced AKI. NLP processes radiology reports and screens data to identify cardiovascular issues, aiding in electronic medical record analysis and workflow improvement.
Seetharam et al., 2021 [15]	United States	Review	ML model	AI is used to determine the appropriateness and strategy of PCI.	Yes	The ML algorithm had better predictive performance for CAD and PCI strategy compared to traditional methods. It analyzes diverse sources, including electronic procedure reports, to identify patients with cardiac conditions. ML also estimates cardiac death risk using adenosine myocardial perfusion data, enabling enhanced risk stratification and outcome prediction.
Samant et al., 2023 [16]	United States	State-of-the-art review	EchoNavigator by Philips	AI is engaged in the pre-procedural planning and clinical phase of the process.	Yes	AI-guided risk assessment models, utilizing ML algorithms, were superior to traditional cardiovascular risk assessment scores in predicting MACE following ACS. Additionally, the study highlighted the potential of AISER technologies to transform pre-procedural planning, device innovation, research and development, regulatory approval, and education and training in cardiovascular interventions.
Molenaar et al., 2022 [17]	Netherlands	Review	ASReview software	AI is utilized for the automated analysis of ICA images, particularly in tasks such as frame selection, segmentation, lesion evaluation, and functional assessment of coronary flow.	Yes	The research incorporated NLP for selecting features through a method known as frequency-inverse document frequency, and a Naïve Bayes classifier was progressively trained to tag documents as either pertinent or irrelevant. AI can streamline lesion detection, optimize diagnostic precision, inform treatment choices, and optimize patient results. However, issues such as bias and interpretability must be tackled, and prospective applications include automated reporting and prognosis prognostication.
Seetharam et al., 2022 [18]	United States	Review	ML model	AI is engaged in the phase of data analysis and interpretation, where machine learning algorithms can extract patterns and insights from large datasets.	Yes	ML algorithms promise better diagnostics in nuclear cardiology, electrophysiology, and heart failure. They enhance imaging like SPECT and CMR, improve risk stratification, and predict outcomes in CAD, HCM, and HF. ML aids early detection of conditions like AF and VT/VF, forecasts treatment responses, and predicts electrical storm occurrences. It emphasizes ML’s benefits, challenges, and the need for medical professionals’ involvement in development. It addresses limitations and highlights AI’s potential to streamline clinical processes and improve patient outcomes.
Ben Ali et al., 2021 [19]	Sweden	Review	N/A	AI is engaged in the phase of data-driven AI systems where complex statistical methodologies are utilized to discover new relationships between inputs, actions, and outcomes.	Yes	The study explores ML and DL in cardiology, emphasizing transparency and ethics while addressing challenges like interpretability and regulatory concerns. It discusses promising unsupervised methods such as variational autoencoders and GANs, as well as ML models aiding diagnosis, risk prediction, and treatment planning, with a focus on improving safety and diagnostic accuracy in interventional cardiology.
Petousis et al., 2023 [20]	Greece	Review	N/A	AI is engaged in the phase of intravascular imaging analysis for quick and accurate coronary plaque characterization, guiding stent implantation, and predicting PTCA results.	Yes	The study discusses techniques like rotational atherectomy, excimer laser, and IVUS/OCT for treating calcified lesions, with AI aiding plaque characterization. Intravascular imaging guides PCI, optimizing outcomes, especially with drug-eluting stents. It emphasizes the importance of understanding and managing calcified lesions, noting the role of intravascular imaging and the exploration of artificial intelligence for better treatment outcomes.
Chowdhury and Osborn 2021 [21]	United States	Review	N/A	The AI application is engaged in the interpretation of large volumes of coronary physiology data, specifically in guiding interventional decisions.	Yes	The study discusses coronary physiological assessment techniques like FFR and iFR in guiding treatment decisions for CAD. FFR-guided revascularization reduces major cardiac events; physiological assessment guides treatment decisions. FFR-guided PCI improves outcomes; AI enhances data interpretation. NHPRs assess coronary artery disease severity.
Chu et al., 2023 [22]	China	Review	DL models	Various applications in the diagnosis, management, and treatment of CAD	Needs more trials	The study applied DL models to CAD tasks, enhancing image assessment, risk prediction, and therapy optimization, like stent evaluation. The study found promising results for DL in optimizing CAD therapy, especially in stent evaluation. Despite challenges like generalization, DL improved CAD diagnosis efficiency and interventional navigation, with the potential to enhance objectivity and automation in cardiovascular imaging.
Ramadani et al., 2022 [23]	-	Review	DL models	3D ultrasound catheter segmentation	N/A	The study examines catheter and guidewire tracking technologies, including EM tracking, shape sensing, robotic ultrasound, image-based tracking, DL, haptic feedback, fiber optic shape sensing, bioelectric navigation, hybrid tracking, and MRI-based methods. It highlights advancements like DL for 3D ultrasound catheter segmentation and real-time MRI, aiming to enhance accuracy and efficiency in cardiovascular and interventional radiology.
Karako et al., 2018 [24]	Japan	Review	Multimodal stacked deep polynomial networks	Diagnosing diseases based on imaging, specifically using NNs to classify pathology images.	Yes	NNs aid risk prediction, detect preterm births, predict colorectal cancer, and diagnose diseases accurately. They're crucial for imaging diagnosis and expected to alleviate physician shortages in specific medical fields.
Calvinho et al., 2008 [25]	Portugal	Review	N/A	Data analysis interprets research on mitral regurgitation surgical management.	N/A	The study explored percutaneous mitral valve repair, annuloplasty, and compared replacement versus repair. It discussed evolving techniques, emphasized further research, and highlighted early intervention’s importance in preventing HF.
Prandi and Antunes​​​​​​​ 2023 [26]	Italy	Review	N/A	Evaluating the structural characteristics of valvular apparatus, including leaflet segmentation, the annular perimeter, and valve area size, for patients with mitral, aortic, and tricuspid valves, is an essential component of the assessment and management of valvular heart disease.	N/A	Multimodal imaging, such as ECG, CT, MRI, and fusion imaging, plays a crucial role in guiding transcatheter interventions for patients with mitral and tricuspid valve disease. The use of AI enhances valvular assessment, aiding in pre-procedural planning and intraprocedural guidance. This results in improved patient outcomes in the management of valvular heart disease.
Kwiecinski et al., 2023 [27]	Poland	Review	Autoplaque software	Provide detailed insights into plaque characteristics and aid in the risk stratification of patients with stable chest pain.	Yes	The study demonstrated the utility of AI tools like Autoplaque® software in accurately quantifying plaque characteristics and identifying high-risk features in patients with CAD, enhancing risk stratification and clinical management. Advanced imaging techniques like 18F-fluoromethylcholine PET/CT and 18F-sodium fluoride PET/CT aid in assessing vessel wall alterations and plaque characteristics in CAD. Combining information from coronary PET imaging and CT angiography using machine-learning modeling optimizes risk stratification and disease progression prediction.

Discussion

AI has been extensively used in cardiovascular medicine, encompassing a range of applications in areas such as robotics, natural language processing (NLP), DL, computer vision, ML, and cognitive computing. Although AI and ML are frequently used together, ML is a collection of methods that enable AI to operate. Specifically, there are three primary techniques in AI and ML: reinforcement learning, unsupervised learning, and supervised learning [14].

AI is typically applied in interventional cardiology through both physical and virtual means. Physical AI techniques involve robotic interventions, whereas virtual AI encompasses a broader range of methods, such as ML, NLP, cognitive computing, and automated clinical decision support systems [15]. The extensive use of AI in electrophysiology has primarily focused on signal interpretation. This field heavily relies on the analysis of signals originating from various types of sensors and technologies. Two common types of signals include photoplethysmography (PPG) and electrocardiography (ECG). In recent years, the incorporation of AI in wearable technologies, such as smartphones, bracelets, and smartwatches, has become increasingly prevalent with the use of PPG technology [12].

Types of AI Applications in Interventional Cardiology

ML is a field of study that allows computers to learn through experience without explicit programming. DL, a subset of ML, uses multiple layers of NN that are similar to the human brain. This algorithm uses processing nodes to establish connections and organizes itself into layers [29]. Human intervention in this algorithm is minimal, and it is usually necessary to make imperative changes.

Azzalini et al. used ML to analyze data from 2648 patients who underwent percutaneous coronary intervention (PCI) to assess the relationship between different contrast agents and acute kidney injury (AKI) [30]. The algorithm did not detect any significant changes; however, this highlights the importance of pattern recognition in large datasets using ML. Ghaffar et al. also demonstrated this in 344 TAVR patients, where semi-supervised ML was used to identify phenotypic groups based on clinical and demographic characteristics and their connection to clinical outcomes [31]. The algorithm distinguished five different groups, with the fifth group having the highest in-hospital and 30-day cardiovascular mortality rates. Predicting mortality outcomes is a function of ML, which was also demonstrated by Hernandez-Suarez et al. in 10,833 patients who underwent TAVR. The models showed in-hospital mortality with an area under the curve (AUC) > 0.80 [32].

Many studies have demonstrated the potential of AI in detecting arrhythmias. Alhusseini et al. designed an ML algorithm that classifies intracardiac electrical patterns during atrial fibrillation (AF) by analyzing electro-anatomical mapping data from biatrial sites using basket catheters [33]. The researchers used a CNN, which is primarily used for the visual aspects of data. Schilling et al. used a fuzzy decision tree algorithm to categorize data from complex fractionated atrial electrograms (CFAEs) based on their characteristics [34]. The algorithm successfully identified four distinct subgroups, achieving an accuracy rate of 81 ± 3%. Furthermore, it displayed 100% accuracy in detecting continuous electrograms. The versatility of this algorithm makes it particularly useful in instances where data are ambiguous, thereby providing a more adaptable alternative to conventional decision trees [34].

Cho et al. utilized DL techniques to characterize coronary plaques, specifically focusing on high-risk plaque features and quantifying calcium extent and fibroatheroma based on IVUS data [35]. Nishi et al. introduced an IVUS-based DL algorithm to measure the lumen, vessel, and stent area, resulting in a smoother stent implantation [36]. Similarly, Min et al. utilized DL to analyze procedural parameters such as stent length, diameter, and dilation pressure, with an algorithm predicting preprocedural stent under-expansion [8]. The Emory University group developed a ML algorithm for IVUS segmentation and calculation of lumen area and plaque burden, demonstrating excellent agreement with expert analysis [14]. DL has also been applied to OCT images. Through analyzing the OCT images, DL algorithms can provide calcium risk scores, which can aid in modifying the lesion and stent placement strategies. These scores can predict stent under-expansion and malposition in calcified lesions undergoing PTCA [20]. As previously mentioned, CNNs are particularly useful for analyzing visual data. Du et al. utilized this algorithm to train on 3990 images and evaluate on 2711 images to detect characteristics of lesions, including diameter stenosis, calcification, thrombus, and dissection [37]. Chu et al. developed a CNN model for plaque characterization in OCT, training the model on a dataset from multiple centers and validating it against expert consensus [22].

Several attempts have been made to improve coronary computed tomography angiography (CCTA). For instance, Ciusdel et al. developed a DL model to detect cardiac phase and end-diastolic frames in coronary angiograms [38]. The model was trained on a substantial dataset comprising 17,800 images from over 3,000 patients and was evaluated on 27,900 angiograms from 6,250 patients. Additionally, Itu et al. utilized a DL model that used geometric features from CCTA as inputs to integrate the nonlinear relationships between various anatomical features [39]. Other studies have implemented AI to reconstruct coronary arteries in three dimensions and assess the hemodynamic significance of stenosis [16]. Moreover, DL has been employed to detect and track catheters and guidewires based on instance segmentation. These approaches enable fully automatic frameworks for catheter tracking by extracting an ordered set of points on the catheter centerline or by converting the problem into two stages: region-of-interest detection and target segmentation. However, ML methods often require a large amount of annotated data, which can be time- and labor-intensive [23].

AI has been widely used in robotics. With ML, robotic navigation systems have been enhanced in precision and safety for catheter ablation procedures. Feasibility studies have demonstrated ML algorithms that guide automated electro-anatomical voltage mapping using remote magnetic navigation systems [12]. These algorithms use previous mapping procedures to learn and improve ablation procedures. In coronary interventions, AI-driven robotics can be used to decrease procedural duration [16]. Additionally, AI has been employed in robotic navigation systems to enhance procedural precision in PCI and peripheral interventions. Algorithms have also been tested as robotic surgical assistants in TAVR. Robotics has also been tested for PCI. The utilization of robotic assistance significantly reduces radiation exposure to cath lab staff compared with manual PCI [16]. In a prior study, a CNN was implemented in a robot-assisted catheterization process [6]. The model provides real-time stent quantification and precise 3D rendering by aggregating information from adjacent OCT frames. Another study demonstrated the use of 2D ultrasound frames for catheter tracking with AI estimating the 3D shape [23]. AI has also employed particle filtering and template matching in this procedure.

Clinical outcomes and efficacy

The integration of AI into the field of cardiology holds great promise for enhancing disease diagnosis and management, ultimately resulting in improved patient outcomes [14]. The utilization of DL in clinical practice has the potential to revolutionize the cardiology community and provide new avenues for enhancing various aspects of interventional processes. AI can play a crucial role in improving the diagnosis of coronary artery disease (CAD) and personalized interventions for individual patients [14]. Early studies have shown that ML algorithms can achieve a high level of accuracy (94%) in predicting myocardial infarction in patients presenting with chest pain in the emergency department [14]. The adoption of AI in clinical practice has far-reaching implications that extend beyond enhancing patient outcomes. AI can play a significant role in congenital heart disease management by enabling early intervention, risk stratification, and customized treatment planning. These approaches can reduce complications and enhance the quality of life of patients with this condition [20]. Additionally, AI can optimize the allocation of resources and improve the cost-effectiveness of healthcare. AI can minimize adverse effects and improve the efficacy of interventions by generating personalized treatment plans tailored to individual characteristics. Furthermore, AI-powered real-time decision support can empower clinicians to make informed choices to improve patient care. These benefits demonstrate the potential of AI in transforming healthcare and improving patient outcomes [20].

Automated analysis of all preoperative mobile and clinical data can provide patient-specific risk scores for procedural planning and yield valuable predictors for postprocedural care [20]. Algorithms can also be applied to patients with cardiogenic shock to determine who may benefit from mechanical circulatory support. DL prediction models can predict the periprocedural risks of death, bleeding, contrast nephropathy, and stroke [20]. Predictive analytics with cognitive computing can support clinical decision-making and assist in prioritizing tasks in catheterization laboratories. By utilizing real-time data and making intraprocedural predictions, adverse events can be avoided. The integration of pre-, intra-, and postprocedural data can aid in monitoring patient recovery, predicting complications, and determining the optimal duration of medical therapy [20]. After discharge, postoperative data from personal devices can be integrated with hospitalization data to optimize patient recovery and reduce readmission rates. Advanced AI tools can be utilized to automate the analysis and interpretation of various imaging modalities such as OCT, IVUS, ECG, CCTA, and magnetic resonance angiography (MRA) [20].

The application of AI-driven robotics in cardiovascular diagnosis and treatment has the potential to streamline coronary interventions, reduce the procedure duration, and enhance patient outcomes. In clinical studies, robot-assisted PCI has been proven to be safe, feasible, and free of radiation exposure and musculoskeletal strain in the coronary arteries, peripheral lower extremity arteries, and carotid arteries [20]. In recent years, DL algorithms have emerged as innovative tools for analyzing vast amounts of data derived from IVUS and OCT for different vessel segments. These algorithms utilize CNNs specifically designed and trained to process large numbers of images. As a result, they can evaluate the data in only a few seconds. Several DL models have been developed for intracoronary imaging, providing a wealth of information that assists the operator during PTCA at various stages [20]. AI has the potential to determine the optimal calcium modification technique or a combination of these, guide stent implantation, and predict the outcomes of PTCA [20]. In a large multicenter observational cohort study, an ML algorithm that combined clinical and computed tomographic variables (including coronary calcium scoring) was found to be superior to conventional cardiovascular risk assessment scores, such as atherosclerotic cardiovascular disease risk scores or calcium scores, in predicting adverse cardiovascular events [16]. The application of AI-guided coronary angiography-based virtual FFR systems can reconstruct coronary arteries in 3D and assess the significance of stenosis [16]. After the stenting procedure, receiving feedback on the degree of stent expansion and hemodynamic function using an AI-based system can assist both the operator and patient in understanding anticipated short- and long-term outcomes [17]. These systems have demonstrated high levels of sensitivity, specificity, positive predictive value, and accuracy compared with invasive FFR [15]. Although AI has made significant advancements in cardiovascular imaging and electrophysiology, its role in intensive care is still in its nascent stage.

Challenges and limitations

The increasing demand for collaborative efforts and data sharing among research institutions presents challenges for data security and privacy [12]. To enhance healthcare through the appropriate utilization of patient data, it is essential to employ data scientists and information technology (IT) professionals under the supervision of hospitals to ensure the protection of patient data. Independent high-quality research is crucial in this regard [19]. It is vital to consider the cybersecurity of hospital information systems that store electronic health records (EHRs). Relying solely on private companies to shape the future of AI in medicine is not the only approach to technological advancement in this field [19]. ML algorithms perform better when trained using specific data; however, when large quantities are used, predictability decreases owing to a lack of generalizability [12]. The ‘black box nature of ML’ phenomenon refers to the situation in which ML algorithms do not provide clear explanations for the conclusions they reach, which can lead to a lack of confidence among clinicians when utilizing ML-based technologies in the process of making critical clinical decisions [12]. The escalating burden of cardiovascular disease is likely to result in an increase in the number of interventions performed, which will consequently lead to an increase in workload and healthcare expenses, as indicated by current cardiology reports [17].

## Conclusions

In conclusion, the integration of AI into cardiovascular medicine - particularly interventional cardiology - marks a paradigm shift with substantial implications for patient care. ML and DL have advanced risk stratification, patient phenotyping, and outcome prediction, notably in procedures such as TAVR. AI-driven image analysis, including CNNs, has enhanced the interpretation of OCT and IVUS, supporting accurate diagnosis and procedural planning. Additionally, AI-powered robotics has improved procedural precision, safety, and efficiency, with demonstrated reductions in radiation exposure and procedure time during robot-assisted PCIs. Despite these benefits, challenges remain, including data privacy concerns and the limited transparency of ML models. Addressing these issues requires interdisciplinary collaboration, regulatory oversight, and expanded data sharing. Enhancing model interpretability and refining predictive algorithms are critical steps toward responsible implementation. With thoughtful integration, AI can enable personalized, evidence-based interventions, reduce complications, and improve outcomes in cardiovascular care.
